# Enhanced and tunable photoluminescence of polyphenylenevinylenes confined in nanocomposite films

**DOI:** 10.1186/s11671-015-0818-2

**Published:** 2015-03-11

**Authors:** Oleg Yu Posudievsky, Mykhailo S Papakin, Oleksandr P Boiko, Vyachesalv G Koshechko, Vitaly D Pokhodenko

**Affiliations:** L.V. Pisarzhevsky Institute of Physical Chemistry of the National Academy of Sciences of Ukraine, prospekt Nauki 31, 03028 Kyiv, Ukraine; Center for Physical Sciences and Technology, Savanoriu 231, LT-02300 Vilnius, Lithuania

**Keywords:** Conjugated polymers, Silica nanoparticles, Nanocomposite films, Spatial confinement, Tunable photoluminescence

## Abstract

Conformation of macromolecules and interchain interactions determine spectral properties of conjugated polymers (CP). An achievement of spatial confinement of isolated chains is one of the routes to use this feature of CP for their purposeful usage. In the present work, CP/O300 nanocomposites based on CP - poly(p-phenylenevinylene) and poly(2-methoxy-5-(2′-ethylhexyloxy)-1,4-phenylenevinylene) - and silica nanoparticles (O300) are prepared. In comparison with many previously known hybrid nanomaterials synthesized with the similar purpose, CP/O300 nanocomposites are characterized by the essentially enhanced and tunable photoluminescence. The greatest change of color coordinates is observed for poly(p-phenylenevinylene)-based nanocomposites due to specific preparation method and interaction with the inorganic component. The main emission from CP in the CP/O300 nanocomposites is owing to 0–0 transitions, while 0–1 transitions, associated with aggregate states of the CP chains, are suppressed.

**Electronic supplementary material:**

The online version of this article (doi:10.1186/s11671-015-0818-2) contains supplementary material, which is available to authorized users.

## Background

Conjugated polymers (CP) attract attention of many researches due to the perspective of their applications in different optoelectronic devices [1-5]. Functional characteristics of such devices depend not only on the chemical structure of the macromolecules but also on their conformation as well as interchain interactions, because π-π interaction can decrease the intermolecular distance and affect the charge transport and energy transfer [4,6,7].

One route to determine the fine details of the processes that control the operation of solid CP-based devices is isolating the polymer chains. Such an effect was achieved previously by extreme dilution of CP solutions in the so-called ‘good solvents’ [8,9], an isolation of CP chains as molecular wires in the structure of polyrotaxanes [10,11] or in a matrix of non-conductive organic polymers [12].

An alternative approach was to achieve confinement of CP in inorganic materials. The effect was achieved when the CP macromolecule is inserted inside the cavities of different 2D and 3D non-conductive porous matrices [13-26]. Such hybrid nanocomposites were prepared by embedding CP macromolecules inside the pre-synthesized mesoporous particles [13-18] and films [19,20], also using an *in situ* method, i.e., during film formation [21,22], in the latter case. The procedure of exfoliation followed by successive adsorption of CP was used for layered matrices [7,23-26].

The studies of confinement of CP macromolecules permitted to establish a number of important regularities for luminescent properties of CP, as well as to evaluate the possibility and advantages of using this effect to create improved optoelectronic devices [7,19,22,23,27].

However, the methods of achieving confinement of CP used previously were often quite complex, and the fraction of CP in the composition of the obtained hybrid nanomaterials was low, or only a portion of macromolecules was in the confinement conditions (the necessity to synthesize the specifically porous inorganic matrices, the low rate of CP macromolecules incorporation inside the pores of the matrix, the probable negative effect of the elevated temperature which is used to promote incorporation on CP, the presence of the sufficient part of CP on the outer surface of the inorganic particles [15,17]).

The present paper considers a new approach to achieve the confinement of CP macromolecules within the films formed by hybrid nanoparticles with a core-shell structure, the core of which consists of non-porous dielectric (silicon dioxide) and the shell is formed by a thin layer of CP chains, and the study of their spectral characteristics. Poly(p-phenylenevinylene) and poly(2-methoxy-5-(2′-ethylhexyloxy)-1,4-phenylenevinylene) characterized by green and red luminescence, respectively, were investigated as examples of CP. The choice of the polymers was based on the desire to study the influence of the preparation process (based on a non-conjugated precursor polymer and soluble conjugated polymer) on the change of their spectral characteristics.

## Methods

### Preparation of the nanocomposites

Poly(p-phenylenevinylene), PPV, prepared from the precursor polymer poly(p-xylene tetrahydrothiophenium chloride) (Aldrich, No. 540765, Sigma-Aldrich, St. Louis, MO, USA) and poly(2-methoxy-5-(2′-ethylhexyloxy)-1,4-phenylenevinylene) (Aldrich, No. 536512, Sigma-Aldrich, St. Louis, MO, USA), MEH-PPV, were used in the work.

Amorphous pyrogenic silica (Orisil300, Orisil, Ltd., Ukraine), O300, was used as an inorganic component of the hybrid nanomaterials. According to [28], it is characterized by a specific area of 300 ± 30 m^2^/g, SiO_2_ portion not less than 99.9%, absence of own porosity, and spherical particle size of 5 to 20 nm.

For preparation of PPV/O300 hybrid nanomaterials, 29 mg of the freshly calcined (8 h at 500°C) O300 was added to 20 mL of an aqueous solution of the precursor polymer (0.25 mg/mL) under constant mechanical stirring. Then, the dispersion was sonicated for 60 min using Sonopuls HD2070 (Bandelin, Electronics, Berlin, Germany) and centrifuged on 5430 centrifuge (Eppendorf, Hamburg, Germany) at a rotation rate of 3,500 rpm. The upper half of the dispersion was used to produce thin films of the hybrid nanocomposite. After deposition of the nanocomposite on a precleaned glass substrate by spin coating (2,500 rpm), PPV/O300 film was prepared by heat treatment at 200°C for 7 h in vacuum (approximately 10^−3^ Torr). An individual PPV film was prepared by a similar route using the solution of the precursor polymer. The formation of the conjugated polymer was verified by the appearance in the IR spectrum of the band at 965 cm^−1^ which corresponds to the vibrations of CH bonds in trans-vinylene groups [29].

For preparation of MEH-PPV/O300 hybrid nanomaterials, 47.6 mg of the freshly calcined O300 was added to 10 mL to the solution of MEH-PPV in toluene (0.5 mg/mL) at constant mechanical stirring. The dispersion was then sonicated for 30 min. The resulting dispersion was used to produce thin films of the hybrid nanocomposite MEH-PPV/O300 coatings by spin coating and subsequent vacuum drying. The films of individual MEH-PPV were prepared analogously using the solution of the polymer in toluene.

The composition of the hybrid nanocomposites was selected so that to yield nanomaterials which could ideally (to exclude the possibility of formation of polymer globules is naturally impossible) have an incomplete monolayer of CP in the space between the adjacent silica nanoparticles. According to the elemental analysis data, the composition of the prepared nanocomposite films SiO_2_/CP was about 10:1 weight ratio.

### Characterization

CHN analysis of CP/O300 nanocomposites was successfully conducted using a Carlo Erba 1106 (Carlo Erba, Milano, Italy) (combustion temperature of 1,030°C, atmosphere of oxygen). TEM images were obtained on a TEM125K (SELMI, Ukraine) microscope working at 100 kV. Amorphous carbon film which covered the copper grid was used as a carrier for samples. UV–vis spectra were measured on double beam spectrophotometer 4802 (UNICO, Fairfield, NJ, USA) with a resolution of 1 nm. Photoluminescence (PL) and Fourier transform infrared spectroscopy (FTIR) spectra were registered using LS55 (PerkinElmer, Waltham, MA, USA) and IFS-66 (Bruker, Karlsruhe, Germany) spectrophotometers. Kinetics of fluorescence decay of the studied materials was measured with the Edinburg Instruments TCSPC Fluorescence Spectrometer F900 (Edinburgh Instruments Ltd., West Lothian, UK) using different lasers for the sample excitation. The fluorescence spectra were corrected for the instrument sensitivity.

## Results and discussion

**Figure 1:**
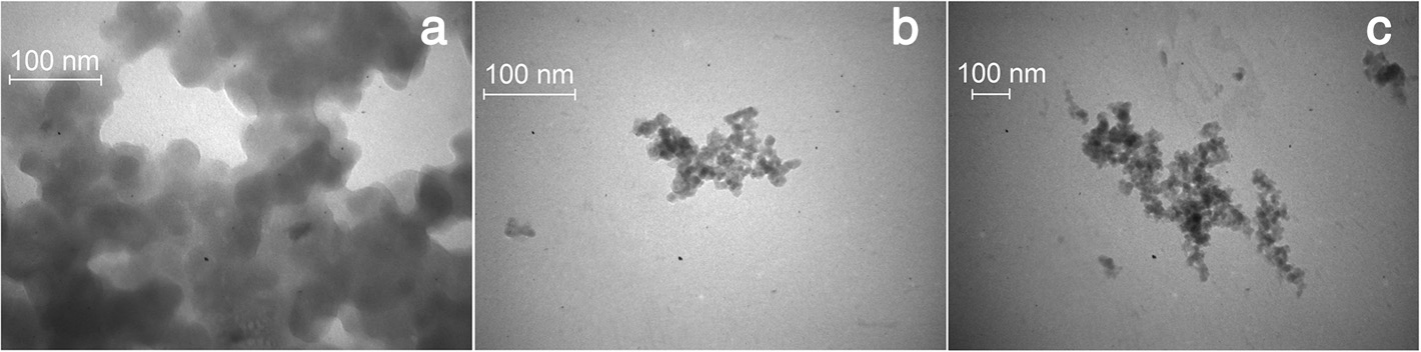
**TEM images of the initial O300 (a) and PPV/O300 (b) and MEH-PPV/O300 (c) nanocomposites.**

It is seen from the TEM data that the particles with a size of 30 to 50 nm (Figure 1a), which are formed as a result of the primary silica nanoparticles (5 to 20 nm) aggregation, were the primary structural element of the pure O300 film. Unlike O300, the size of the particles in the hybrid CP/O300 films was approximately 15 nm (Figure 1b,c). The observed decrease in the size of the nanoparticles was probably due to addition of CP which improves the dispersion of the inorganic nanoparticles in comparison with the initial O300.

**Figure 2:**
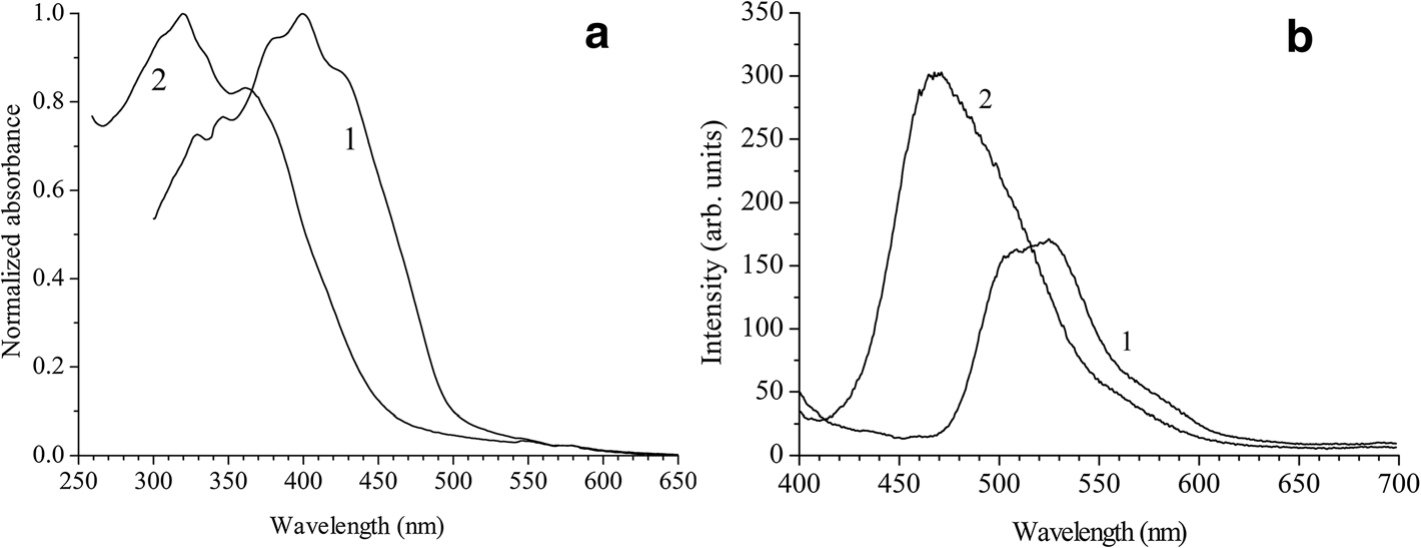
**UV–vis and PL spectra of the individual PPV and PPV/O300 nanocomposite films. (a)** UV–vis and **(b)** PL (*λ*_ex_ = 320 nm) spectra of PPV (1) and PPV/O300 (2).

UV–vis and PL spectra of the individual PPV and PPV/O300 nanocomposite films are shown in Figure 2. As follows from Figure 2a, the maximum of UV–vis spectrum is blue shifted by 80 nm (Figure 2a) at the transition from individual PPV to PPV/O300 nanocomposite. That corresponds to an increase of the optical gap in PPV from 2.36 to 2.48 eV (Additional file 1: Figure S1). The observed shift is due to a significant decrease of conjugation length [9] in the polymer chains in PPV/O300 nanocomposite, in comparison with the pure PPV, due to the influence of the inorganic nanoparticles on the conformation of the macromolecules. PL spectrum of the individual polymer corresponds to the literature data [30-32], has a maximum at 530 nm, and is characterized by a distinct vibronic structure (Figure 2b). PL spectrum of PPV/O300 nanocomposite is consistent with UV–vis spectra and indicates significant differences in comparison with the initial polymer (Figure 2b): (1) the maximum of the spectrum is blue shifted up to 467 nm similarly to UV–vis spectrum (Figure 2a); (2) the integrated PL intensity increases by a factor of 2 (taking into account the renormalization of the PL spectra due to the different optical density of the compared PPV/O300 and PPV films); (3) the vibronic structure is less explicit and, unlike individual PPV, 0–0 transitions are characterized by the maximum intensity; and (4) the spectral width increases in 120 meV.

**Figure 3:**
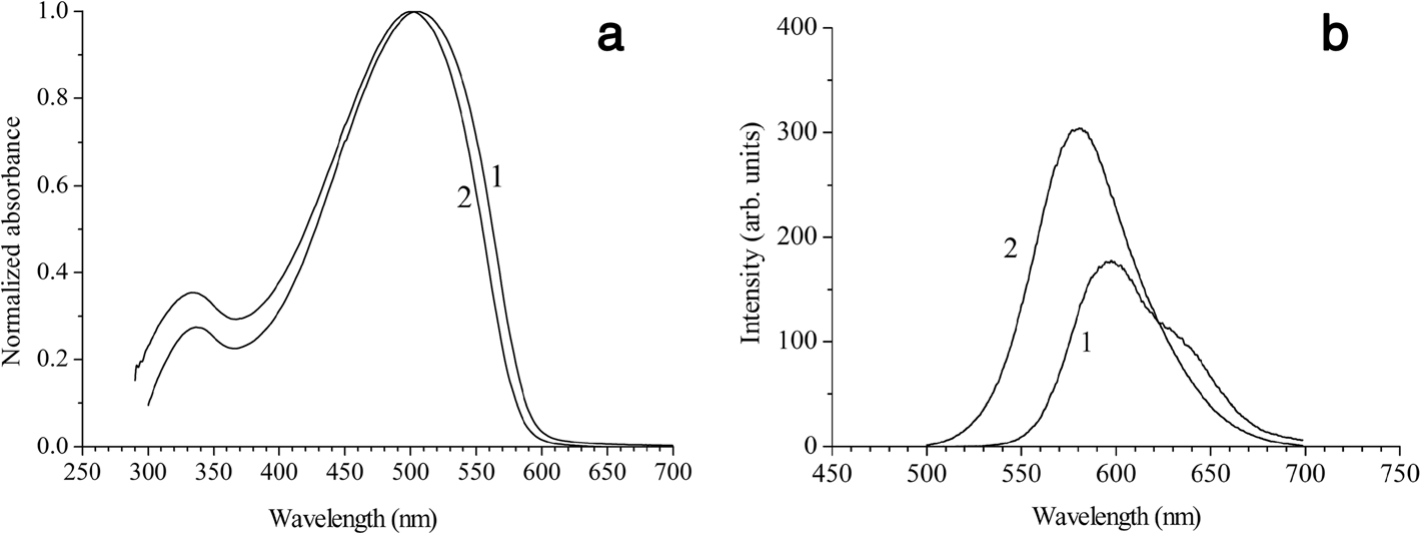
**UV–vis and PL spectra of the individual MEH-PPV and MEH-PPV/O300 nanocomposite films. (a)** UV–vis and **(b)** PL (*λ*_ex_ = 440 nm) spectra of MEH-PPV (1) and MEH-PPV/O300 (2).

UV–vis and PL spectra of the individual MEH-PPV and MEH-PPV/O300 nanocomposite films are shown in Figure 3. It follows from Figure 3 that the maximum in UV–vis spectrum of the MEH-PPV/O300 nanocomposite is shifted relative to the spectrum of MEH-PPV insignificantly in the blue region. The observed shift equal to 4 nm corresponds to the increase of the optical gap of MEH-PPV from 2.04 to 2.06 eV (Additional file 1: Figure S2). This change (0.02 eV) is much less than in the case of the PPV based materials (0.12 eV). The PL spectrum of MEH-PPV film possesses a maximum at 598 nm analogously to [33-35], as well as a characteristic vibronic structure (Figure 3b). In comparison with MEH-PPV, the PL spectrum of the MEH-PPV/O300 nanocomposite is blue shifted by 18 nm that several times exceeds the shift of the absorption spectra (0.02 and 0.06 eV, respectively) and is characterized 1.7 times increased integral PL intensity (taking into account the renormalization of the PL spectra due to the different optical densities of the compared MEH-PPV/O300 and MEH-PPV films) and the width broadened by 83 meV, as well as a significantly less pronounced vibronic structure.

Thus, the presented results testify to the presence of several common feature characteristics of all prepared CP/O300 nanocomposites: the hypsochromic shift of the peak position, the growth of the integral intensity, the weakening of the vibronic structure, and the increased width.

The hypsochromic shift in the PL spectra of the CP/O300 nanocomposites is caused by a decrease in the effective conjugation length in CP chains [9], since it cannot be due to changes in the chemical structure of the polymers as the nanocomposites were prepared without using high temperatures or strong chemicals which could affect their chemical structure. Such a decrease is probably owing to changes in chain conformation due to the confinement of CP between adjacent silica nanoparticles, as well as probable interactions of CP with the surface of O300. A distinctive feature of the CP from other photoluminescent compounds is the strong dependence of their spectral and physical-chemical characteristics on the conformation of the polymer [9,36]. Therefore, it is logical to assume that as a result of the formation of hybrid nanocomposite films, the conformation of macromolecules, in comparison with the individual CP films, varies and conjugation is maintained only in relatively shorter chain segments which determine the spectral properties of the nanocomposites.

The observed blue shifts in the PL spectra of CP/O300 correspond to isolation of CP macromolecules, assuming that the excitons cannot migrate to the regions of polymer chains with a lower energy. This peculiarity is an inherent characteristic of the hybrid nanocomposites based on CP and mesoporous silica or layered inorganic matrices, which were reported in the previous literature [13-19,21,24-26].

At the same time, it should be noted also that the maximum of the absorption spectra of the prepared MEH-PPV-based nanocomposite is red shifted relative to the PL spectra of the diluted polymer solution - 570 nm for MEH-PPV (PPV is insoluble) (Additional file 1: Figure S3), that is consistent with the theory of confinement on the basis of molecular orbitals [15,37,38] which predicts an increase in the energy of all molecular orbitals in confined CP, the highest occupied orbital being more sensitive compared to the lowest unoccupied orbital.

Since the polymer macromolecules in the space between adjacent inorganic nanoparticles have a degree of aggregation smaller than in the individual polymer film, this could cause a change in the shape of the spectra presented in Figures 2 and 3, which consists in reducing the relative intensity of 0–1 transitions. In the case of MEH-PPV, for example, as follows from the numerical fitting of the experimental data the results of which are shown in Additional file 1: Figure S4 and Table S1, the value of Huang-Rhys factor, *S*, which describes the strength of the exciton-phonon interaction in the molecule, decreases in the transition from the individual polymer to the nanocomposite from 0.299 to 0.026. Previously, such effect was observed for CP at low temperatures [39]. In our case, all studies were conducted at room temperature. Therefore, the observed effect is obviously a consequence of fixing the torsional modes due to the spatial confinement of the chains in CP/O300 nanocomposites.

The decrease in aggregation of CP chains in the prepared hybrid nanocomposites has another consequence. Due to the isolation of the macromolecules in the space between the inorganic nanoparticles, the probability of H-aggregates, which in comparison with J-aggregates are characterized by an increased probability of photoexcitation energy dissipation into heat [40], is reduced, which leads to an increase in PL intensity of the CP/O300 nanocomposites relative to CP (Figures 2 and 3). It should be marked that the used content of CP in the prepared nanocomposites (approximately 10%) is not optimal in terms of achieving the maximum intensity of PL. We believe it could be increased thus supporting the macroscopic charge transport when the nanocomposite layer will be used as a functional layer of the device.

**Figure 4:**
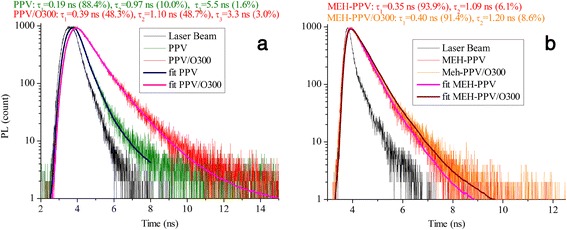
**Comparison of the PL lifetime of (a) PPV and PPV/О300 (** ***λ***_ex_ **= 300 nm) and (b) MEH-PPV and MEH-PPV/О300 (** ***λ***_ex_ **= 470 nm).**

It should be also mentioned that the increase of PL intensity of the CP-based nanocomposites relative to initial polymers agrees with the growth of the PL lifetime that could be seen in Figure 4 which presents the results of measuring the PL decay kinetics. The same tendency was earlier found for MEH-PPV/MCM-41 composite prepared by direct insertion of the CP inside the nanochannels of the mesoporous inorganic matrix [18].

**Figure 5:**
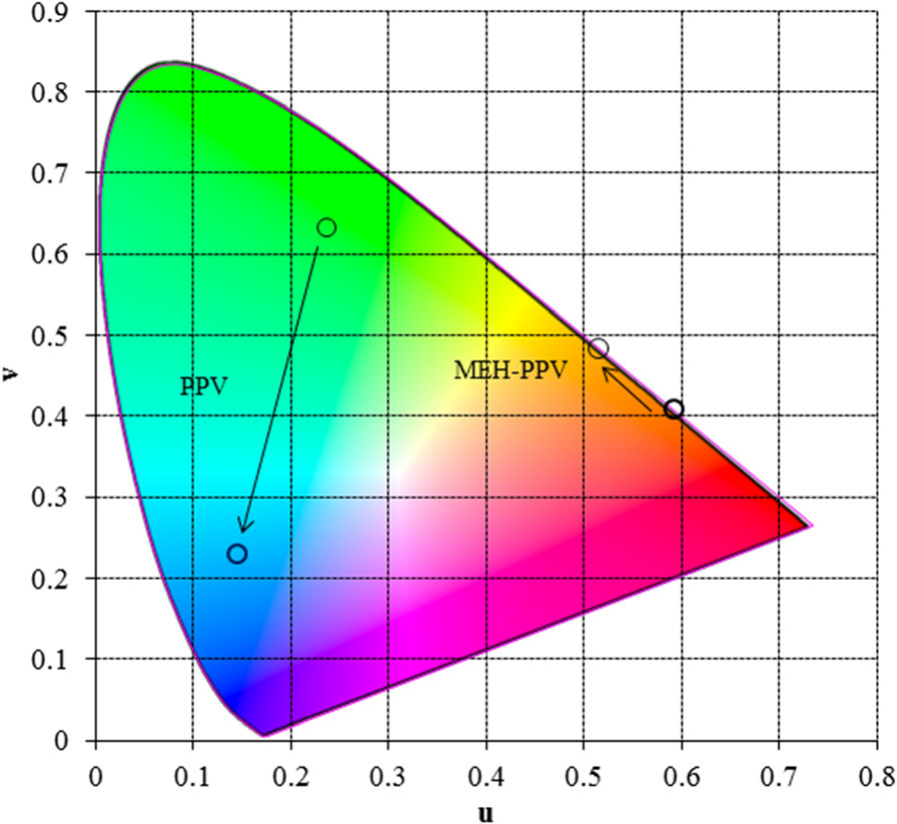
**Color coordinates for PL of the studied CP and CP/O300 nanocomposites.**

The hypsochromic shifts observed in the PL spectra of CP/O300 nanocomposites, in comparison with the PL spectra of the individual CP, lead to substantial changes in color coordinates (*u*, *v*) (Figure 5), which were calculated on the basis of the spectra presented in Figures 2 and 3. As follows from the data shown in Figure 5, the change of the emitted color for PPV-based materials occurs from (0.235, 0.634) to (0.145, 0.231), so that the color of PL changes from green to blue. This determines the possibility of using PPV/O300 nanocomposites for replacement of polyfluorenes in optoelectronic devices.

Unlike PPV, the color change for MEH-PPV-based materials occurs from (0.591, 0.408) to (0.514, 0.248), and its value is small (Figure 5).

It should be noted that the maximum spectral change among the prepared nanocomposites CP/O300 (relative to the initial CP) is observed for the PPV-based nanocomposite. This could be due to the fact, in contrast to other nanocomposites, PPV/O300 was synthesized from the precursor polymer which does not possess continuous conjugation and leads to a higher degree of chain flexibility than other CP. Therefore, during the formation of the nanocomposite in this case, there are more degrees of freedom to change the conformation of the precursor-polymer macromolecules, which subsequently influence the conformation and hence the spectral characteristics of the resulting PPV/O300. Furthermore, the precursor polymer is a salt, its chains are positively charged, which suggests the possibility of a significant electrostatic interaction with the nanoparticles of O300 in an aqueous dispersion.

These features also determine the possibility of formation of chemical bonds between the organic and inorganic components of PPV/O300 nanocomposite: a heat treatment process with elimination of tetrahydrothiophene groups of the precursor polymer could result in formation of Si-O-C bonds, which in turn lead to reduction of the effective conjugation length in the macromolecules and influence the spectral characteristics in accordance with the obtained spectral data. The appearance of the band about 880 cm^−1^ in the FTIR spectrum of the nanocomposite confirms such possibility (Additional file 1: Figure S5).

The difference in the preparation method of PPV from MEH-PPV and hence the method for preparation of the corresponding nanocomposites is also evident in the changes of the width of PL spectra for the CP/O300 nanocomposites. Indeed, as noted above, the width of the PL spectra of all CP/O300 nanocomposites is greater than the width of the spectra of the corresponding individual polymers, the main contribution to the broadening provides the states with higher energy, and the value of the broadening increases in transition from MEH-PPV (83 meV) to PPV (120 meV). The broadening of the PL spectra is due to conformational disorder, and therefore, this relationship coincides with increasing flexibility of the stated CP chains.

## Conclusions

Thus, in the present work, the CP/O300 nanocomposites based on CP, such as PPV or MEH-PPV, and silica nanoparticles, wherein the CP macromolecules are in conditions of the spatial confinement, were prepared. Relatively high level of CP content in the nanocomposites was achieved. In comparison with many previously known hybrid nanomaterials synthesized with the similar purpose, CP/O300 nanocomposites are characterized by the presence of essentially larger changes in the PL spectra - the growth of the integral intensity, hypsochromic shift of the maximum, the weakening of the vibronic structure, the broadening of the spectra - in comparison with the spectra of the corresponding polymers. This fact favors the higher homogeneity of CP distribution in the nanocomposites. It is shown that the main emission of CP in the CP/O300 nanocomposites is due to 0–0 transitions, while PL with lower energy (0–1 transitions), associated with aggregate states of the CP chains, is suppressed. Furthermore, the PL spectra of the nanocomposites are broadened compared with the spectra of the individual polymers due to conformational disorder. Increase of the PL intensity of the CP/O300 is accompanied by the growth of the PL lifetime. It is established that the spectral changes are more significant in the case of using the water-soluble precursor polymer for preparation of the nanocomposites (PPV/O300).

## Additional file

Additional file 1: Figure S1. Evaluation of the optical gap in PPV (a) and PPV/O300 (b). **Figure S2.** Evaluation of the optical gap in MEH-PPV (a) and MEH-PPV/O300 (b). **Figure S3.** Normalized PL spectrum of the diluted solution of MEH-PPV. The data for PPV are absent due to its insolubility. **Figure S4.** Fitting of PL spectra of MEH-PPV (a) and nanocomposite MEH-PPV/О300 (b). **Figure S5.** FTIR spectra of PPV (blue) and PPV/O300 (red). **Table S1.** Parameters of numerical fitting of PL spectra of MEH-PPV and MEH-PPV/О300 using three Gauss curves according to equation: $={y}_0+\varSigma i\Big({A}_i/\left(\sqrt{\frac{\pi }{2}}\;{w}_i\right)\times \exp \left(-2{\left(\left(E\hbox{-} {E}_{ci}\right)/{w}_i\right)}^2\right)$.
