# Supplementary material for: Enhanced and tunable photoluminescence of polyphenylenevinylenes confined in nanocomposite films
Source: Nanoscale Res Lett. 2015 Mar 11;10:118. doi: 10.1186/s11671-015-0818-2 (PMC4385242; doi:10.1186/s11671-015-0818-2)
Supplement: Additional file 1: Figure S1. — Evaluation of the optical gap in PPV (a) and PPV/O300 (b). Figure S2. Evaluation of the optical gap in MEH-PPV (a) and MEH-PPV/O300 (b). Figure S3. Normalized PL spectrum of the diluted solution of MEH-PPV. The data for PPV are absent due to its insolubility. Figure S4. Fitting of PL spectra of MEH-PPV (a) and nanocomposite MEH-PPV/О300 (b). Figure S5. FTIR spectra of PPV (blue) and PPV/O300 (red). Table S1. Parameters of numerical fitting of PL spectra of MEH-PPV and MEH-PPV/О300 using three Gauss curves according to equation: $={y}_0+\varSigma i\Big({A}_i/\left(\sqrt{\frac{\pi }{2}}\;{w}_i\right)\times \exp \left(-2{\left(\left(E\hbox{-} {E}_{ci}\right)/{w}_i\right)}^2\right)$=y0+Σi(Ai/π2wi×exp−2E‐Eci/wi2. [file 11671_2015_818_MOESM1_ESM.pdf]

Supplementary Information

Enhanced and tunable photoluminescence of polyphenylenevinylenes confined in nanocomposite films

O. Yu. Posudievsky,[1]* M. S. Papakin,[1] O. P. Boiko,[2] V. G. Koshechko,[1] V. D. Pokhodenko[1]

[1] *L.V. Pisarzhevsky Institute of Physical Chemistry of the National Academy of Sciences of Ukraine, prospekt Nauki 31, Kyiv 03028, Ukraine. Tel/Fax: +38044 5256672; E-mail: posol@inphyschem-nas.kiev.ua*

[2] *Center for Physical Sciences and Technology, Savanoriu 231, LT-02300, Vilnius, Lithuania. E-mail: oleksandr.boiko@gmail.com*

Tauc plots are used to evaluate the optical gap in the considered materials:

$$(A \cdot E)^{1/2} \sim (E - E_g),$$

where A – absorption, E – energy, E_g – optical gap.

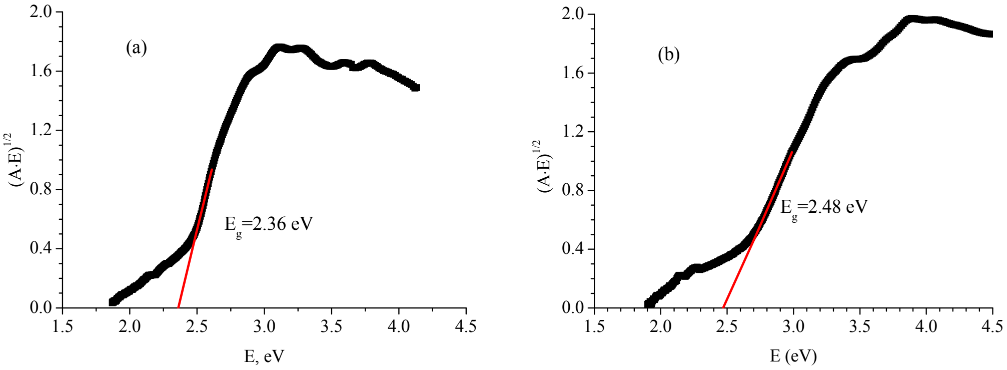

Fig. S1. Evaluation of the optical gap in PPV (a) and PPV/O300 (b).

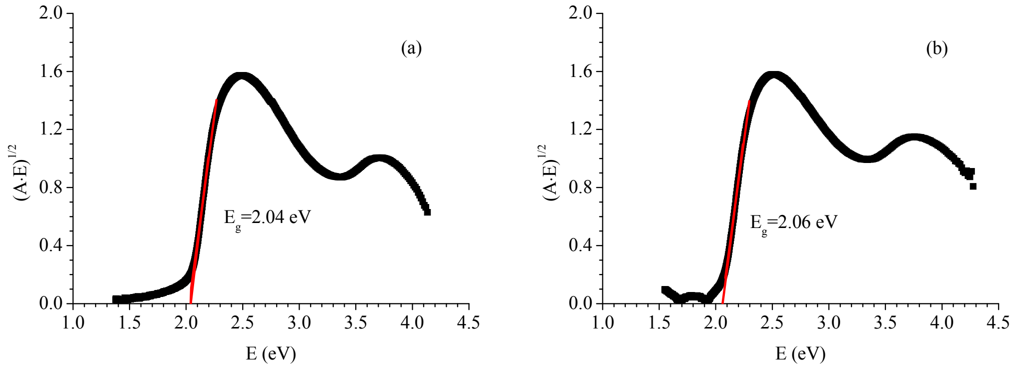

Fig. S2. Evaluation of the optical gap in MEH-PPV (a) and MEH-PPV/O300 (b).

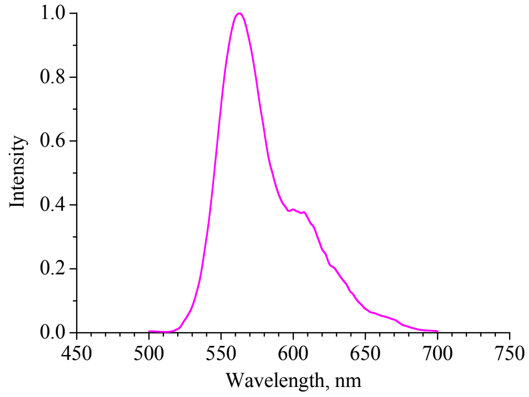

Fig. S3. Normalized PL spectrum of the diluted solution of MEH-PPV. The data for PPV are absent due to its insolubility.

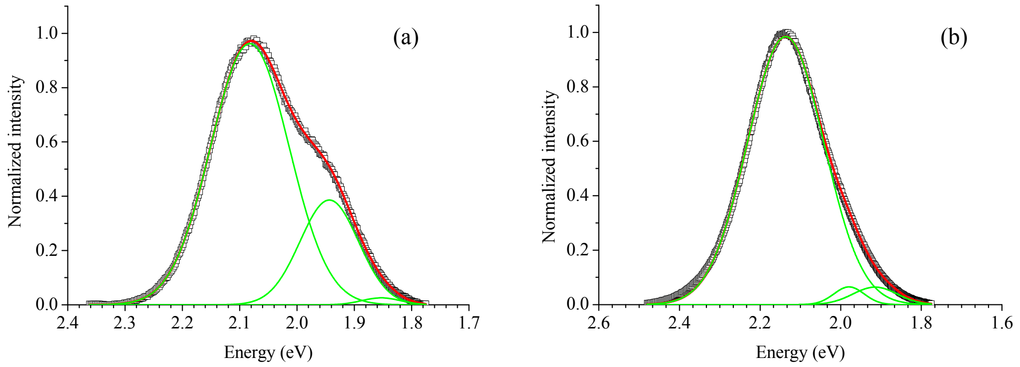

Fig. S4. Fitting of PL spectra of MEH-PPV (a) and nanocomposite MEH-PPV/O300 (b).

Table S1. Parameters of numerical fitting of PL spectra of MEH-PPV and MEH-PPV/O300 using three Gauss curves according to equation: $y=y_0+\Sigma_i(A_i/(\sqrt{\frac{\pi}{2}}w_i)\times\exp(-2((E-E_{ci})/w_i)^2)$.

Parameters	MEH-PPV	MEH-PPV/O300
y_0	0±0	0±0
E_{c1}	2.08232±0.00037	2.13665±0.00027
w_1	0.13633±0.00046	0.18877±0.00054
A_1	0.16498±0.00081	0.23351±0.0006
E_{c2}	1.94369±0.00059	1.97949±0.00663
w_2	0.10133±0.00196	0.07332±0.01235
A_2	0.04932±0.00138	0.00608±0.00463
E_{c3}	1.85311±0.00593	1.91497±0.02893
w_3	0.06626±0.00756	0.10855±0.02374
A_3	0.00216±0.00072	0.00882±0.00469
R^2	0.99975	0.99923
$S=\frac{A_2}{A_1}$	0.299	0.026

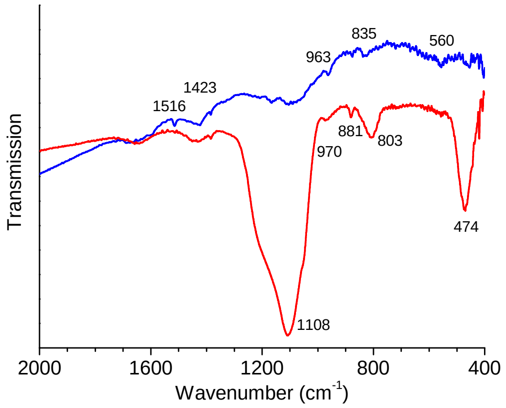

Fig. S5. FTIR spectra of PPV (blue) and PPV/O300 (red).

FTIR spectrum of PPV contains the bands which are characteristic of this conjugated polymer: 560, 835, 963, 1423 and 1516 cm^{-1} [S1, S2]. The bands about 474, 803 and 1108 cm^{-1}, which are characteristic of SiO_2 [S3, 17], dominate in the spectrum of PPV/O300 nanocomposite. The spectrum of the nanocomposite also contains the bands about 801 cm^{-1} and 970 cm^{-1}. The band about 801 cm^{-1} corresponds to vibrations of Si–O–C bonds [S4], whereas the band about 970 cm^{-1} is due to vibrations of the bonds in the trans-vinylene groups of the polymer [S1, S2].

References

[S1] D. M. Byler, Y. Patel, G. A. Arbuckle-Keil. An IR study of poly-1,4-phenylenevinylene (PPV), the 2,5-dimethoxy derivative [$(MeO)_2$-PPV], and their corresponding xanthate precursor polymers and monomers. *Spectrochim. Acta A*, 2011, **79**, 118–126.

[S2] D. D. C. Bradley, R. H. Friend, H. Lindenberger, S. Roth. Infra-red characterization of oriented poly(phenylene vinylene). *Polymer*, 1986, **27**, 1709–1713.

[S3] M. Nakamura, Y. Mochizuki, K. Usami, Y. Itoh, T. Nozaki. Infrared absorption spectra and compositions of evaporated silicon oxides (SiO_x). *Solid State Commun.* 1984, **50**, 1079–1081.

[S4] T. Oh, C. K. Choi. Comparison between SiOC Thin Films Fabricated by Using Plasma Enhance Chemical Vapor Deposition and SiO_2 Thin Films by Using Fourier Transform Infrared Spectroscopy. *J. Korean Phys. Soc.* 2010, **56**, 1150–1155.
